# Affordability of an NGO-government partnership for community-based disability rehabilitation

**DOI:** 10.4102/ajod.v12i0.1283

**Published:** 2023-12-22

**Authors:** Kelsey R. Vaughan, Ram K. Thapa

**Affiliations:** 1 Bang for Buck Consulting, Amsterdam, The Netherlands; 2 The International Humanitarian Action Program, University of Warsaw, Warsaw, Poland

**Keywords:** health economics, cost per DALY averted, disability-adjusted life years, community-based rehabilitation, affordability, health expenditure, disability, UNCRPD

## Abstract

**Background:**

Tunafasi is a community-based rehabilitation (CBR) programme for persons with disability, implemented by a local non-governmental organisation in Uvira, Democratic Republic of Congo, in partnership with government. To assess affordability and support discussions with the government about continued financing and implementation, Tunafasi representatives commissioned a cost-effectiveness study of the programme’s health component.

**Objectives:**

This study aimed to estimate the programme’s impacts, costs, cost per disability-adjusted life year (DALY) averted and affordability of the health component implemented from February 2019 to December 2021.

**Method:**

Health-related improvements were assessed for a sample of 511 persons with disability and converted to DALYs averted. Total expenditure during the period February 2019 to December 2021 was estimated from audited financial statements. The cost per DALY averted was estimated by dividing total programme expenditure by the sum of DALYs averted and compared against newly generated, country-specific thresholds to assess affordability.

**Results:**

The programme cost $55 729.00 to implement from February 2019 to December 2021 and averted 234 DALYs in 511 persons, at a cost per DALY averted of $224.00. This falls above the affordability threshold of $54.00 – $199.00.

**Conclusion:**

While the cost per DALY averted is higher than what thresholds consider affordable for Democratic Republic of Congo, improved engagement from CBR facilitators and greater possibilities for treatment in the post-pandemic era should improve results.

**Contribution:**

This new CBR implementation modality offers a possibly affordable solution to African governments struggling to operationalise disability commitments such as United Nations Convention on the Rights of Persons with Disabilities.

## Introduction

The World Report on Disability estimated that 15% of the global population, equating to around one billion persons, live with some form of disability, 80% of whom are in low- and middle-income countries (LMICs) (World Health Organization [WHO] 2011). These persons with disabilities (PWDs) have long-term physical, mental, intellectual or sensory impairments which, in interaction with various barriers, may hinder their full and effective participation in society on an equal basis with others (United Nations n.d.). Despite the progress made in recent years, PWDs continue to face discrimination and stigma on the grounds of disability, a lack of access to physical environments, assistive devices and essential rehabilitation services, and a lack of support for independent living (WHO 2018). They are often excluded from education, health, and employment and other aspects of society, leading to an increased risk of poverty (WHO 2011).

Following the Declaration of Alma-Ata in 1978, the WHO endorsed the concept of community-based rehabilitation (CBR) to enhance the quality of life of PWDs through multisectoral disability prevention and rehabilitation. Since 2006, CBR is guided by a matrix across five components: health, education, livelihood, empowerment and socialisation. Under each component, there are five elements, each implemented through specific activities (WHO 2010).

Democratic Republic of Congo (DRC) is a low-income country in sub-Saharan Africa. An estimated 10.5 million (11%) of the country’s 96 million population have one or more disabilities, the result of disease and illness, genetic and chromosomal conditions, poverty and a weak healthcare system as well as ongoing conflict (Street Child 2018; WHO 2011). The government of DRC has taken several measures to protect PWDs, including adopting a disability policy in 2010, ratifying the United Nations Convention on the Rights of Persons with Disabilities in 2015, enshrining the rights of PWDs in the Constitution and endorsing a CBR strategy, which is to be implemented by the Ministry of Health by health zones (Ministry of Health n.d.). Despite these developments, ongoing internal conflict, corruption and other challenges have limited the government’s ability to provide accessible and affordable services for PWDs, and various non-governmental organisations (NGOs) such as Humanity and Inclusion, International Committee of the Red Cross, and Fédération Nationale des Associations Des Personnes Vivant Avec Handicap Du Congo (FENAPHACO) have stepped in to help fill the gap, though these service offerings are often fragmented in nature (Street Child 2018).

In the Uvira health zone of South Kivu province of eastern DRC, Appui au Développement de l’Enfant en Detresse (ADED), a local NGO, has been facilitating the implementation of a disability prevention and rehabilitation programme under the name ‘Tunafasi’ in partnership with the local health zone and with initial financial support from a private foundation in the Netherlands. The programme’s overall aim is to enable a positive environment, sustainable autonomy and equal opportunity for PWDs under the age of 33 years and their communities through the integration of the CBR programme in the state system and with community contributions. The programme is based on the CBR matrix and modelled after the Inspire2Care disability prevention and rehabilitation programme, implemented by the Karuna Foundation in Nepal, a Nepali NGO (Karuna Foundation Nepal n.d.). Tunafasi has brought together a number of organisations and service centres that previously provided fragmented services for PWDs in Uvira, including Sosame (mental health services), Centre Bethanie (physical rehabilitation), Haki Yetu, Center d’éducation et formation intégré (CEFI) and Center la Providence des Sourds d’Uvira (CEPROS) and ADED in a network called Muungano Tegemeza. With initial support from Liliane Fonds, ADED and network partners worked together to expand the range of services offered to encapsulate activities across all five CBR pillars (Muungano Tegemeza Network 2023). Together with 22 CBR facilitators (CBRFs), who are government employees with a background in nursing and work at 22 different health facilities, ADED officers facilitate the implementation of the programme (ADED 2020).

Disability prevention activities focus on strengthening maternal and child healthcare in the public health centres through training and equipment support and creating demand for services through awareness raising. Rehabilitation activities are based on the CBR matrix and are customised to address the unique needs of each PWD. Upon their identification, each PWD is assessed by a multidisciplinary team of doctors, physiotherapists, psychologists and other experts, after which an individual rehabilitation plan is jointly developed together with the PWD, his or her family and the CBRF. With the support from ADED officers, the CBRFs make home visits, provide counselling to PWDs and their families, provide physiotherapy or orient parents on it, facilitate medical treatment of PWDs from local health centres, hospitals or referral service centres and link them with local resources. Medical rehabilitation may include counselling, physiotherapy, medical treatment, assistive devices, nutrition support and/or referral medical services. The rehabilitation elements under other pillars range from tuition fee support, home-based education, self-help groups, vocational training, saving and credit activities and more. The programme equally works to strengthen the public system (health zone, health centre, schools) as well as the community support system like self-help groups, organisations of PWDs and child clubs. Community-based rehabilitation facilitators are currently paid by government, while ADED officers and other programme costs are supported by private investors from the Netherlands. There are plans to fully transition programme funding and implementation to the government and the community within 5 years (ADED 2020). With the start of the Tunafasi programme, Uvira became the only health zone in the country known to be implementing all five CBR pillars (though as of early 2023 Tunafasi has now expanded to Baraka and Goma health zones). Six other health zones in South Kivu are known to be implementing the health pillar only; for the country as a whole, 37 out of the country’s 517 health zones are known to be implementing the health pillar only.

Globally, the coverage of CBR is currently very low and there is a lack of available evidence about its effectiveness and cost-effectiveness (Iemmi et al. 2013), partially because of methodological challenges related to evaluating across five distinct domains (Shumba, Haufiku & Mitonga 2020). There are also perceptions that health services for PWDs are unaffordable for governments, though methods do exist for assessing the cost, cost-effectiveness and affordability of the health component, particularly medical rehabilitation services (United Nations n.d.; Vaughan & Thapa 2015).

As part of efforts to encourage rollout of the CBR strategy in other government health zones, Tunafasi donors commissioned a cost-effectiveness evaluation and affordability assessment of the first 2 years of implementation of the programme’s medical rehabilitation component. The assessment aimed to generate evidence to support advocacy efforts with the government health zone and community structures about the affordability of medical rehabilitation integrated in the primary healthcare system.

## Research methods and design

### Study objective

This study aimed to assess the impacts, costs and cost per disability-adjusted life year (DALY) averted of the health component of the Tunafasi programme (medical rehabilitation services) implemented from February 2019 to December 2021, to support discussions with the government and potential long-term partners about its long-term affordability.

### Study setting

Uvira health zone is one of 34 health zones in the South Kivu province of Eastern DRC. Uvira City is the largest metropolitan area, with around 350,000 persons and 22 health centres, and is located at the extreme north end of Lake Tanganyika and linked by roads to Bukavu, the capital of South Kivu province, and to Bujumbura, the economic capital of Burundi. Assuming 11% of the population has a disability, there are an estimated 38,500 persons living in Uvira with a disability.

### Study design

This retrospective study of the Tunafasi programme was modelled after similar studies conducted in Nepal (Vaughan & Thapa 2015). We assessed costs over a start-up period of 11 months (February 2019–December 2019), and costs, benefits measured in DALYs averted and the cost per DALY averted of the medical rehabilitation services delivered during the first 2 years of implementation (January 2020 – December 2021). We took a programme (implementer) perspective, meaning only costs and benefits incurred by the implementers (ADED and the government) are included.

### Sample

We took a purposive sample of the total number of PWDs identified by ADED as of 31 December 2021 (*n* = 1200), aiming to include only PWDs who had been receiving medical CBR-related services from ADED and/or government CBRFs. The final sample selected was based on data availability: there were 764 PWDs that had been assessed and a rehabilitative plan developed; some had only recently been assessed or were deemed to have died, been lost to follow-up or represented duplicate records. The final sample included 511 PWDs for whom complete information was available.

### Programme data collection and analysis

ADED staff provided qualitative information about PWD in the sample, including their diagnosed disability(ies) and subtype(s), using standard categories taken from the Global Burden of Disease (GBD) 2019. Researchers verified the diagnosed disability(ies) against other information provided about the PWD, such as narrative descriptions of their condition(s) and functional limitations and photos. Once confirmed, researchers assigned a disability weight (DW) to each diagnosis. Disability weights are standardised values set by the GBD based on multicountry survey data and represent the loss of health on a scale of ‘0’ (perfect health) to ‘1’ (equivalent to death) (Table 1) (Institute for Health Metrics and Evaluation n.d.). Global Burden of Disease provides no DW for ‘autism’, so we assumed a weight of 0.011. In the case of multiple disability types and/or subtypes for a single person, we summed the weights for each diagnosis and then verified that the combined weight represented the burden caused by the conditions. Summed weights that exceeded 1 or were deemed too high were reduced.

**TABLE 1:** Disability types, subtypes and disability weights.

Type of disability	Subtype for each disability (GBD)	Definition (GBD)	DW
Physical	Disfigurement_mild	Has a slight, visible physical deformity that others notice, which causes some worry and discomfort.	0.011
	Disfigurement_moderate	Has a visible physical deformity that causes others to stare and comment. As a result, the person is worried and has trouble sleeping and concentrating.	0.067
	Disfigurement_severe	Has an obvious physical deformity that makes others uncomfortable, which causes the person to avoid social contact, feel worried, sleep poorly, and think about suicide.	0.405
	Motor_mild	Has some difficulty in moving around but is able to walk without help. May include partial paralysis.	0.01
	Motor_moderate	Has some difficulty in moving around, and difficulty in lifting and holding objects, dressing and sitting upright, but is able to walk without help. May include partial paralysis.	0.061
	Motor_severe	Is unable to move around without help, and is not able to lift or hold objects, get dressed or sit upright. May include partial and/or full paralysis.	0.402
Mental and psychosocial	Depression_mild	Feels persistent sadness and has lost interest in usual activities. The person sometimes sleeps badly, feels tired, or has trouble concentrating but still manages to function in daily life with extra effort.	0.145
	Depression_moderate	Has constant sadness and has lost interest in usual activities. The person has some difficulty in daily life, sleeps badly, has trouble concentrating, and sometimes thinks about harming himself (or herself).	0.396
	Depression_ severe	Has overwhelming, constant sadness and cannot function in daily life. The person sometimes loses touch with reality and wants to harm or kill himself (or herself).	0.658
	Schizophrenia	Schizophrenia, acute and/or residual state. Acute: hears and sees things that are not real and is afraid, confused, and sometimes violent. The person has great difficulty with communication and daily activities, and sometimes wants to harm or kill himself (or herself). Residual: hears and sees things that are not real and has trouble communicating. The person can be forgetful, has difficulty with daily activities, and thinks about hurting himself (or herself).	0.683
	Autism†	Some degree of difficulty with social interaction and communication. May exhibit atypical patterns of activities and behaviours, such as difficulty with transition from one activity to another, a focus on details and unusual reactions to sensations.	0.011
Intellectual	Borderline intellectual functioning	Is slow in learning at school. As an adult, the person has some difficulty doing complex or unfamiliar tasks but otherwise functions independently.	0.011
	Intellectual disability and mental retardation, mild	Has low intelligence and is slow in learning at school. As an adult, the person can live independently, but often needs help to raise children and can only work at simple supervised jobs.	0.043
	Intellectual disability and mental retardation, moderate	Has low intelligence and is slow in learning to speak and to do even simple tasks. As an adult, the person requires a lot of support to live independently and raise children. The person can only work at the simplest supervised jobs.	0.1
	Intellectual disability and mental retardation, profound	Has very low intelligence, has almost no language and does not understand even the most basic requests or instructions. The person requires constant supervision and help for all activities.	0.2
	Intellectual disability and mental retardation, severe	Has very low intelligence and cannot speak more than a few words, needs constant supervision and help with most daily activities, and can do only the simplest tasks.	0.16
Vision-related	Complete blindness	Is completely blind, which causes great difficulty in some daily activities, worry and anxiety, and great difficulty going outside the home without assistance.	0.187
	Blindness	Is blind in one eye and has difficulty judging distances.	0.017
	Low vision	Vision loss which cannot be corrected with glasses, contacts or surgery. Limited sight remains but person may experience blind spots, poor night vision and blurry sight, which causes difficulty in daily activities, some emotional impact (e.g. worry), and some difficulty going outside the home without assistance.	0.184
Hearing related	Deaf	Complete hearing loss: cannot hear at all in any situation, including even the loudest sounds, and cannot communicate verbally or use a phone. Difficulties with communicating and relating to others often cause worry, depression or loneliness.	0.215
	Hearing loss, adult onset: mild	Has great difficulty hearing and understanding another person talking in a noisy place (e.g. on an urban street).	0.01
	Hearing loss, adult onset: moderate, untreated	Is unable to hear and understand another person talking in a noisy place (e.g. on an urban street) and has difficulty hearing another person talking even in a quiet place or on the phone.	0.027
	Hearing loss, adult onset: severe or profound, untreated	Is unable to hear and understand another person talking, even in a quiet place, is unable to take part in a phone conversation, and has great difficulty hearing anything in any other situation. Difficulties with communicating and relating to others often cause worry, depression and loneliness.	0.204
Vocal and speech related	Vocal and speech related	Has difficulty speaking, and others find it difficult to understand.	0.051

*Source:* Institute for Health Metrics and Evaluation, n.d., *Global health data exchange,* viewed May 2023, from https://ghdx.healthdata.org/record/ihme-data/gbd-2019-disability-weights

GBD, global burden of disease; DW, Disability weight.

† , Assumed DW since condition does not exist in GBD.

ADED also provided information on the services or treatments received, a narrative description of the health-related changes in each PWD and a classification of their improvement compared to their baseline diagnosis, all as of 31 December 2021. We classified improvement using a standard scale which ranged from ‘0’ to ‘4’, with ‘0’ corresponding to no change in health status (i.e. same as baseline disability diagnosis), ‘1’ representing mild improvement, ‘2’ reflecting moderate improvement, ‘3’ reflecting significant improvement and ‘4’ reflecting that the PWD had been deemed by ADED and/or government CBRFs to be fully rehabilitated. A score of ‘4’ was only given in a few cases where the impairment has been medically and/or surgically corrected, for example, a vision-related issue that was resolved with glasses. In other cases, the maximum possible score was a ‘3’ to recognise the continued presence of the disability despite treatment. In a few cases, negative improvements were seen, meaning the condition worsened; for example, the PWD suffered increased epileptic attacks despite taking epilepsy medication. Here a ‘0’ was recorded on the assessment improvement scale. Researchers cross-referenced narrative descriptions of the starting health state and treatments received with the description of the noted improvement and the improvement scale score to ensure consistency.

The improvement scale score was then translated to a change in DW. For each one-point assessment improvement scale increase (e.g. from ‘1’ to ‘2’, or from ‘2’ to ‘3’), the DW was reduced by 25%. For example, a PWD with ‘hearing loss, adult onset: severe or profound, untreated’ received hearing aids and was deemed to have achieved an improvement of ‘3’. The starting DW of 0.204 was reduced by 0.153 (0.75 × 0.204), resulting in a new DW of 0.051 (0.204 – 0.153). This approach is in line with some DWs themselves, where different DWs are available for different levels of severity of the condition and/or treated and untreated states.

The change in DW was then used to calculate DALYs averted. DALYs are a measure of overall disease burden, expressed as the number of years lost due to ill-health, disability (meaning reduced capacity due to disease) or early death. Medical interventions including rehabilitation can avert DALYs and increase the number of years that a person lives in good health (Anon n.d.). We used the prevalence formula DALY = YLL + YLD, where YLL is the years of life lost and YLD is the years lost due to disability. Years of life lost is assumed to be zero, and YLD = I × DW × L, where I is the number of incident cases, DW is the change in disability weight and L is the life expectancy in years (Fox-Rushby & Hanson 2001). Life expectancy refers to the duration the benefit will be sustained without further investment. For individuals deemed fully rehabilitated, no further services are needed; therefore, the duration of benefit is the individual’s remaining lifetime (Vaughan, Thapa & Paudel 2018). For all other interventions, it was assumed the benefits were to last the programme’s lifetime (5 years); this was not adjusted to account for the exact remaining programme lifetime as the date of the intervention was unknown. Remaining life expectancy from the time of improvement (December 2021) was calculated assuming the average life expectancy at birth for each individual (Vaughan et al. 2018), based on the World Bank data for DRC, minus years of life already lived (their age) as of end 2021 (The World Bank n.d.).

We attributed 100% of the claimed DALYs averted to the Tunafasi programme even though some expenditures (such as donated medical services or assistive devices) which contributed to averting these DALYs have been excluded from the expenditure calculations.

### Cost data collection and analysis

We reviewed programme records, audited financial statements and other financial records and extracted relevant information about expenditures related to medical rehabilitation services during the study period (February 2019 – December 2021) into a Microsoft Excel analysis file created for this study. Expenditures which were related to multiple CBR pillars or shared across pillars (education, livelihood, social and empowerment) were allocated to the health component as a percentage share of health expenditures of all five CBR pillar expenditures, calculated from expenditure records. Expenses not related to the health pillar of CBR were excluded. Costs incurred by patients (e.g. for travel), their families (e.g. caregivers’ time) or other organisations (e.g. donated devices) were excluded.

Expenditures exclusively used for the health pillar of CBR were allocated 100%. Shared expenses (such as office costs, salaries of staff working on both health and other CBR pillars or non-CBR-related activities) were apportioned to the health pillar on the basis of percentages suggested by advisors to the programme.

Finally, we adjusted expenditures to reflect the share of the sample of the total population (511/1200), because the total incurred expenditures also reflect the assessment costs incurred for PWDs who are not yet receiving services (i.e. *n* = 1200 − 511 = 689). This adjustment assumes that PWD assessed but who had not yet received services and PWD in the sample incurred the same average expenditure per person, as we have no basis to know the differential in average per person cost between PWD only assessed but not yet receiving services and PWD assessed and receiving services.

Expenditures deemed to be related to CBR’s health pillar were then classified into four groups: (1) human resources (including ADED staff and CBRFs salaries which are paid by the government), (2) implementation (meaning direct programme costs), (3) running costs (such as office expenditures and bank charges) and (4) other. The ‘other’ category encompasses technical assistance (TA) from project advisors, costs related to developing partnerships with other disability-related programmes and the government, and learning-related costs such as conferences. Expenditures associated with preparatory activities for the programme (incurred in 2019), and other investment-type expenditures such as training, whose benefits extended over multiple years, were annualised across the programme years (until end 2024) using a 3% discount rate, and then only the annualised expenditures falling inside the study period were included. Expenditures were converted from Euros to US dollars ($) using the average exchange rate for 2019–2021 (1 Euro = $0.87) (The World Bank n.d.).

### Cost per disability-adjusted life year averted

Cost per DALY averted was calculated as the adjusted annualised intervention costs from February 2019 to December 2021 divided by the number of DALYs averted in the sample.

### Ethical considerations

This study used existing financial records and anonymised participant data from ADED’s monitoring and evaluation database. No ethical approval was required.

## Results

### Sample characteristics

The sample of 511 PWDs was 45.0% female and 54.8% male, with one respondent classified as ‘other’. It included PWDs up to the age of 33 years; females had an average age of 15.0 years, males 13.8 years, and the sample as a whole 14.4 years.

In terms of number of disabilities classified per person, 332 PWDs had a single disability (65%), 147 exactly two disabilities (29%), and 32 exactly three disabilities (6%). Physical disabilities overwhelmingly dominated the sample (Table 2).

**TABLE 2:** Types of disability present in the sample.

Type of disability	Primary disability		Secondary disability		Tertiary disability	
	*n*	%	*n*	%	*n*	%
Hearing-related	23	5	12	7	0	0
Intellectual	10	2	5	3	0	0
Mental and psychosocial	38	7	11	6	4	12
Neurological	34	7	9	5	2	6
Physical	350	68	130	73	20	61
Vision-related	34	7	3	2	2	6
Vocal and speech-related	22	4	9	5	5	15

**Total**	**511**	**100**	**179†**	**100**	**33**	**100**

† , Includes the 147 with exactly two disabilities and 32 with exactly three disabilities.

### Disability-adjusted life years averted

In the sample (*n* = 511), a total of 249.34 DALYs were averted, an average of 0.49 DALYs per person. This reflects an average improvement of just over one point (1.32) on the improvement scale, indicating a move, for example, from ‘mild’ to ‘moderate’ improvement. However, there were a large number of PWDs (*n* = 118) who saw no improvement. Of these, 83 (70%) had not yet received medical rehabilitation services, received services and saw no improvement or refused medical services. This means their improvement score was 0 and therefore 0 DALYs were averted. Removing these 83 from the sample, we see a slight increase in the average improvement score and average DALYs averted per person, from 1.32 to 1.58, representing 0.58 DALYs averted per person.

### Cost findings

The total cost of the health component of the CBR programme was $130 870.000 over the period February 2019 to December 2021. Over two-thirds (64%) of expenditures were for direct programme implementation, around one-fifth (22%) were for staff, 8% were for running costs and 7% were classified as ‘other’ (Table 3).

**TABLE 3:** Health-related community-based rehabilitation costs by category, 2019–2021 ($).

Category	2019	2020	2021	2019–2021	
				*n*	(%)
Human resources	9026	11 370	8071	**28 467**	**(22%)**
Implementation	4938	24 304	54 344	**83 586**	**(64%)**
Running costs	1744	2594	5914	**10 252**	**(8%)**
Other	997	1994	5576	**8566**	**(7%)**

**Total**	**16 705**	**40 261**	**73 905**	**130 870**	**-**
**(%)**	**(13%)**	**(31%)**	**(56%)**	**-**	**(100%)**

Note: ‘Implementation’ refers to direct programme costs. ‘Running costs’ include office expenditures, bank charges, etc. ‘Other’ encompasses TA from The Netherlands, partnership development costs and learning-related costs such as conferences.

When adjusted to reflect only expenditures corresponding to the sample, the total annualised expenditures for the period 2019–2021 are $55 729.00.

### Cost per disability-adjusted life year averted

Given the adjusted expenditures ($55 729.00) and number of DALYs averted for the sample (249.34), the cost per DALY averted is $224.00.

## Discussion

The study evaluated the economic efficiency of a newly implemented collaboration between regional health authorities and a local NGO to improve the provision of medical rehabilitation services for PWDs in Uvira, DRC. This study evaluated the costs, DALYs averted and cost per DALY averted of the programme to assess the feasibility of long-term continuation of the programme without external funding as part of the government’s primary healthcare system.

This study estimated that the programme spent $55 729.00 to assess and provide medical rehabilitation services to 511 PWDs, which resulted in 234 DALYs averted at a cost per DALY averted of $224.00. There is little research done on the cost per DALY averted of medical rehabilitation services for PWDs which can offer a comparison. A previous study was conducted in Nepal of a similar disability rehabilitation programme, which found a cost per DALY averted of €384.00 – €843.00 (approx. $335.00 – $735.00) (Vaughan et al. 2018). However, this assessment used earlier GBD DWs and included more adults, for whom fewer DALYs are often averted due to their shorter remaining lifespans. Therefore, it is hard to directly compare the two studies.

Previously, it was common to use a threshold defined as three times gross domestic product (GDP) per capita as a guide to determining the cost-effectiveness of health interventions. However, this specific threshold approach has fallen out of use after multiple criticisms over its failure to consider local resource availability (Bertram et al. 2016; Leech et al. 2018; Robinson et al. 2016). Two alternatives to assess affordability include comparing against a benchmark intervention already offered by the health system (but there are no known comparators for DRC), and country-specific cost per DALY averted thresholds that reflect health opportunity costs. There are currently two such thresholds for DRC. Ochalek and colleagues used estimated elasticities on mortality, survival, morbidity and DALYs, while also taking into account infrastructure, changes in donor funding, country-specific data on health expenditure, epidemiology and demographics, to estimate a threshold of $54.00 – $69.00 per DALY averted (2015 US$, approx. $62.00 – $79.00 in 2021), which is only 12% – 15% of the GDP per capita for DRC (Ochalek, Lomas & Claxton 2018). A similar approach was used by Daroudi and colleagues, who used evidence from the GBD study, per capita health expenditure, human development index (HDI) score and GDP per capita for 176 countries over the period 2000 to 2016 (Daroudi et al. 2021). They found the threshold cost per DALY averted was, on average, 0.34 times the GDP per capita in low HDI countries like DRC. This would equate to an approximate threshold of $199.00, based on the 2021 GDP per capita of $584, in our setting. Our cost per DALY averted estimate of $224 is above both thresholds, but still lower than roughly half of other interventions implemented in LMIC settings (Horton 2018). It is also important to note that while cost-effectiveness and affordability are important from an economic standpoint, value of particular interventions and their incorporation in, for example, essential health benefit packages guaranteed by government is normally guided by a wider range of criteria, among others, burden of disease, equity and feasibility (Hayati et al. 2018; Horton 2018). Guaranteeing these services despite their cost per DALY averted falling above affordability thresholds would be a timely operationalisation of the June 2022 law which aims to better promote and protect the rights of PWDs (UNCRPD Fund n.d.).

Although some exceptional cases will likely still require outside support to treat in the future, this study has shown that the majority of PWDs can receive medical rehabilitation services from government in a cost-effective way. The innovative implementation of this programme, through the government’s primary healthcare system and with initial, limited-term external financial support from investors in the Netherlands, offers a promising alternative to the more traditional model of fragmented rehabilitation activities implemented by various NGOs, often in parallel with government efforts. However, lobbying and advocacy efforts with the government are needed to ensure a financial commitment to fully finance medical rehabilitation when external support ends, which can be secured by tapping into government funding envelopes specifically available for vulnerable populations. Health spending in DRC is among the lowest in the world, both as a total ($19.69 per capita in 2018 [2020 US$]) and in terms of government’s contribution ($2.62 per capita [13%]) (The World Bank n.d.). Given the population of Uvira (387 421), the Tunafasi programme costs approximately $0.14 per person per year, of which the government’s share is presently 5.3% or less than $0.01 per person per year. Local authorities can already start taking over an increasing share of costs of and responsibility for the programme by increasing the level of effort of the part-time CBRFs and providing them with necessary training and financial as well as non-financial motivation.

Community-based rehabilitation programmes are traditionally challenging to evaluate, namely because of their breadth across health, education, livelihoods, empowerment and social aspects (Hartley 2016). Our study has focused on costs and health-related gains of medical rehabilitation services, given the existence and suitability of the DALY as a metric for measuring improvements as a result of medical rehabilitation. We also chose to focus on medical rehabilitation given the need to assess the long-term affordability of the programme in terms that can be easily understood by the Ministry of Health, given that CBR is housed in the Ministry of Health in DRC and the DALY is the most common outcome measure for health services in LMICs. However, we have not captured some of the major benefits stemming from CBR programmes’ four other pillars, such as increased community mobilisation around disability, skills gained by the members of families of PWDs, improvements in attitudes of community members towards PWDs and social cohesion. This is an important limitation of our study (Vaughan et al. 2018).

## Conclusions

Medical rehabilitation services can be affordable for government in LMIC settings such as DRC and represent good value for money at a cost per DALY averted of $224.00. The implementation and funding model presented in this article, through the government’s primary healthcare system and with initial external financial support from investors, can be considered for replication in other LMICs.
